# Pravastatin activates activator protein 2 alpha to augment the angiotensin II-induced abdominal aortic aneurysms

**DOI:** 10.18632/oncotarget.15104

**Published:** 2017-02-04

**Authors:** Hui Ma, Wen-Jing Liang, Mei-Rong Shan, Xue-Qing Wang, Sheng-Nan Zhou, Yuan Chen, Tao Guo, Peng Li, Hai-Ya Yu, Chao Liu, Ya-Ling Yin, Yu-Lin Wang, Bo Dong, Xin-Yan Pang, Shuang-Xi Wang

**Affiliations:** ^1^ Key Laboratory of Cardiovascular Remodeling and Function Research, Qilu Hospital, Shandong University, Jinan, China; ^2^ Department of Pediatrics and Department of Cardiology, Shandong Provincial Hospital Affiliated to Shandong University, Jinan, China; ^3^ College of Pharmacy and School of Basic Medical Sciences, Xinxiang Medical University, Xinxiang, China; ^4^ Department of Neurology, The Peoples Hospital of Xishui County, Huangang, Hubei, China; ^5^ Hubei Key Laboratory of Cardiovascular, Cerebrovascular, and Metabolic Disorders, Hubei University of Science and Technology, Xianning, China

**Keywords:** abdominal aortic aneurysm, pravastatin, AMP-activated protein kinase, activator protein 2 alpha, matrix metalloproteinase 2, Pathology Section

## Abstract

We have previously reported that activation of AMP-activated kinase alpha 2 (AMPKa2) by nicotine or angiotensin II (AngII) instigates formation of abdominal aortic aneurysms (AAA) in *Apoe*−/− mice. Statins, used to treat hyperlipidemia widely, activate AMPK in vascular cells. We sought to examine the effects of pravastatin on AAA formation and uncover the molecular mechanism. The AAA model was induced by AngII and evaluated by incidence, elastin degradation, and maximal abdominal aortic diameter in *Apoe*−/− mice. The phosphorylated levels of AMPKa2 and activator protein 2 alpha (AP-2a) were examined in cultured vascular smooth muscle cells (VSMCs) or in mice. We observed that pravastatin (50 mg/kg/day, 8 weeks) remarkably increased the AngII-induced AAA incidence in mice. In VSMCs, pravastatin increased the levels of pAMPK, pAP-2a, and MMP2 in both basal and AngII-stressed conditions, which were abolished by tempol and compound C. Pravastatin-upregulated MMP2 was abrogated by AMPKa2 or AP-2a siRNA. Lentivirus-mediated gene silence of AMPKa2 or AP-2a abolished pravastatin-worsened AAA formations in AngII-infused *Apoe*−/− mice. Clinical investigations demonstrated that both AMPKa2 and AP-2a phosphorylations were increased in AAA patients or human subjects taking pravastatin. In conclusion, pravastatin promotes AAA formation through AMPKa2-dependent AP-2a activations.

## INTRODUCTION

Abdominal aortic aneurysms (AAAs) are permanent dilations of the abdominal aorta with over 85% mortality after rupture. There are no therapeutic strategies but surgery proven to blunt AAA progression and rapture. Pathologically, AAA is characterized by dilatation of all layers of the arterial wall as a result of loss of elastin, vascular smooth muscle cell (VSMC) apoptosis, and compensatory collagen deposition [1–3]. For example, angiotensin II (AngII) increases matrix metalloproteinases (MMPs) to induce AAA formation in animal experiments by upregulating oxidative stress [4, 5].

Statins are cholesterol-lowering drugs and widely used in the treatment of hypercholesterolemia to prevent the development of atherosclerosis through improving endothelial function [6–8]. However, statins produce several adverse effects, such as insulin resistance [9], skeletal muscle toxicity and myocardial atrophy [10]. The AMP-activated protein kinase (AMPK), consisting of catalytic α subunit and regulatory subunits β and γ, has a pivotal function in energy homoeostasis in eukaryotes. AMPK is activated by pravastatin in skeletal muscle and endothelial cells [11, 12]. We reported that AMPK activation mediated AngII-induced AAA formation in *Apoe*^−/−^ mice [13]. This alludes that pravastatin is potentially to promote AAA incidences.

Activator protein 2α (AP-2α) is a member of the AP-2 transcription factor family consisting of α, β, γ, δ, and ε subunits [14]. Mice deficient in AP-2 die after birth due to the abnormal development, suggesting an important role of AP-2 in mammals [15, 16]. Park *et al* reported that nerve growth factor stimulated endothelial cell invasion by augmenting MMP-2 via AP-2α-dependent gene transcription, which may be responsible for triggering angiogenesis [17]. We found that aspirin activates AMPK to increase AP-2α in accelerated atherosclerotic plaque [18]. This study is aimed to investigate the effects of pravastatin on AAA formations. Our results demonstrate that pravastatin augments AngII-induced AAA incidence through AMPKα2/AP-2α signaling.

## RESULTS

### Pravastatin promotes the incidence and severity of AAA formation in AngII-infused *Apoe*^−/−^ mice

**Figure 1:**
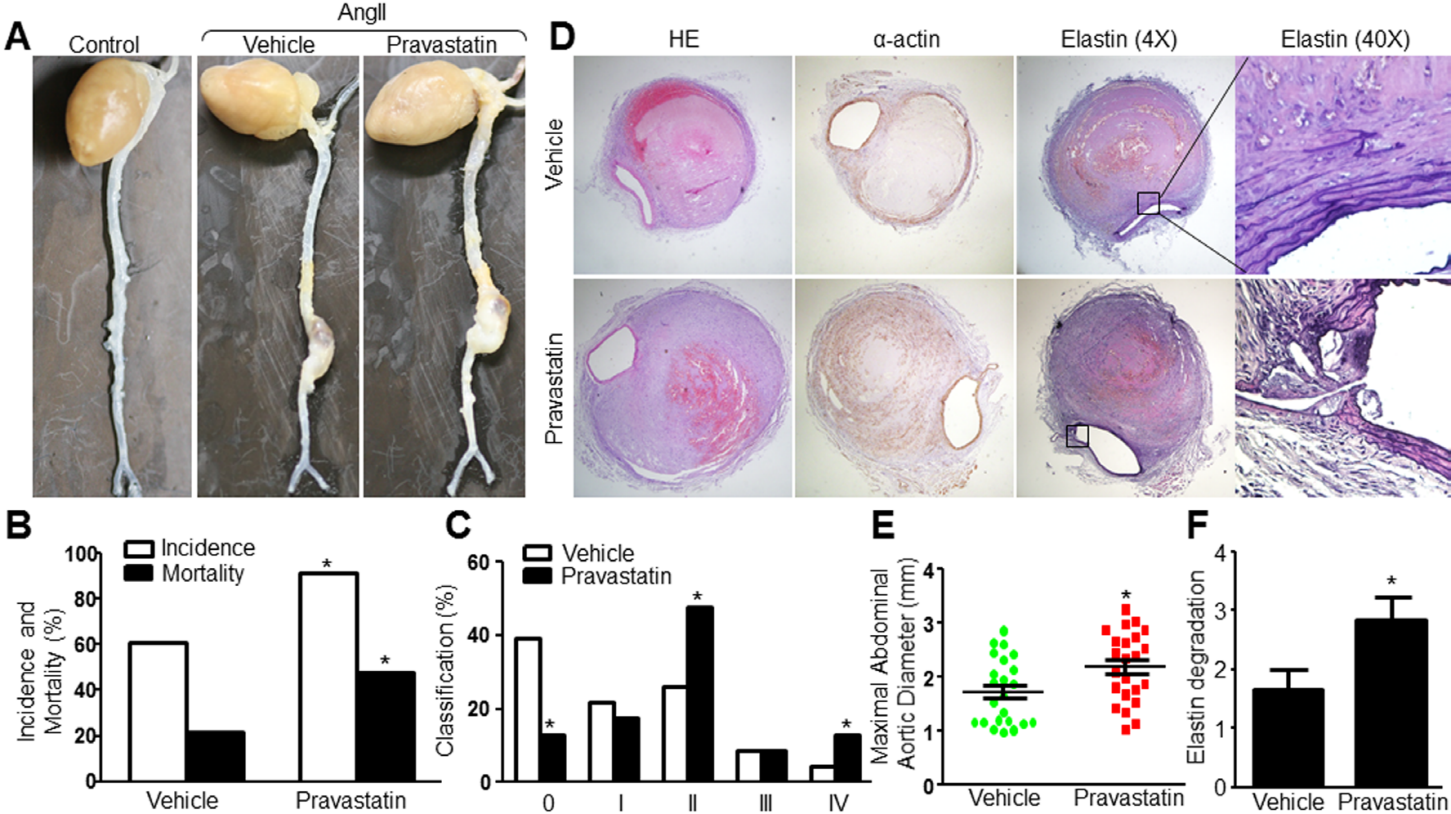
Pravastatin promotes AngII-induced AAA formation in *Apoe*−/− mice *Apoe*−/− mice were received pravastatin administration (50 mg/kg) in drinking water for 4 weeks followed by AngII infusion (1.44 mg/kg/day) for another 4 weeks. **A**. Representative images showing macroscopic features of abdominal aortic specimens in mice. **B**. The incidence and the mortality, and **C**. the severity of AAA in AngII-infused mice. **D**. Histological analysis of HE staining, immunohistochemistry of α-actin, and Verhoff-Van Gieson staining in abdominal aortic cross-section. **E**. Maximal abdominal aortic diameters and **F**. grades of elastin degradation in aneurysmal tissues. N is 15-23 in each group. Chi-Square test was used for statistical comparisons in **B**. and **C**. Unpaired student's *t*-test was used for statistical comparisons in E and F. *P < 0.05 *vs*. Vehicle.

As reported [19, 20], we found that treatment with AngII (1.44 mg/kg/day) for 28 consecutive days promotes AAA formation in *Apoe*−/− mice as evidenced by enlarged abdominal aortas morphologically (Figure 1A). To determine the effects of statin on AAA formation, we treated AngII-infused *Apoe*−/− mice with pravastatin (50 mg/kg/day) for 8 weeks. Compared with mice treated with vehicle, pravastatin significantly increased the AAA incidence (60.87% *VS* 91.30%, *P*<0.05), the mortality and the severity of AAA in AngII-infused mice (Figure 1B and 1C). Histological analysis also indicated that the maximal aortic diameter and the degradation of elastin were increased by pravastatin in *Apoe*−/− mice with AAA (Figure 1D-1F). These results suggest that pravastatin promotes the AngII-induced AAA formation in *Apoe*−/− mice.

We also detected whether pravastatin affected hemodynamic parameters or metabolic indexes in AngII-infused *Apoe*−/− mice. As indicated in Supplementary Table S1 and S2, pravastatin treatment had no effects on heart rates, blood pressures, plasma triglyceride and glucose levels in AngII-infused *Apoe*−/− mice. Interestingly, the levels of total cholesterol and LDL cholesterol, but not HDL cholesterol, were increased by pravastatin in *Apoe*−/− mice, demonstrating that the effect of pravastatin on AAA formation is not due to the lipid-lowering effects.

### Pravastatin via ROS increases AMPK phosphorylation in VSMCs

**Figure 2:**
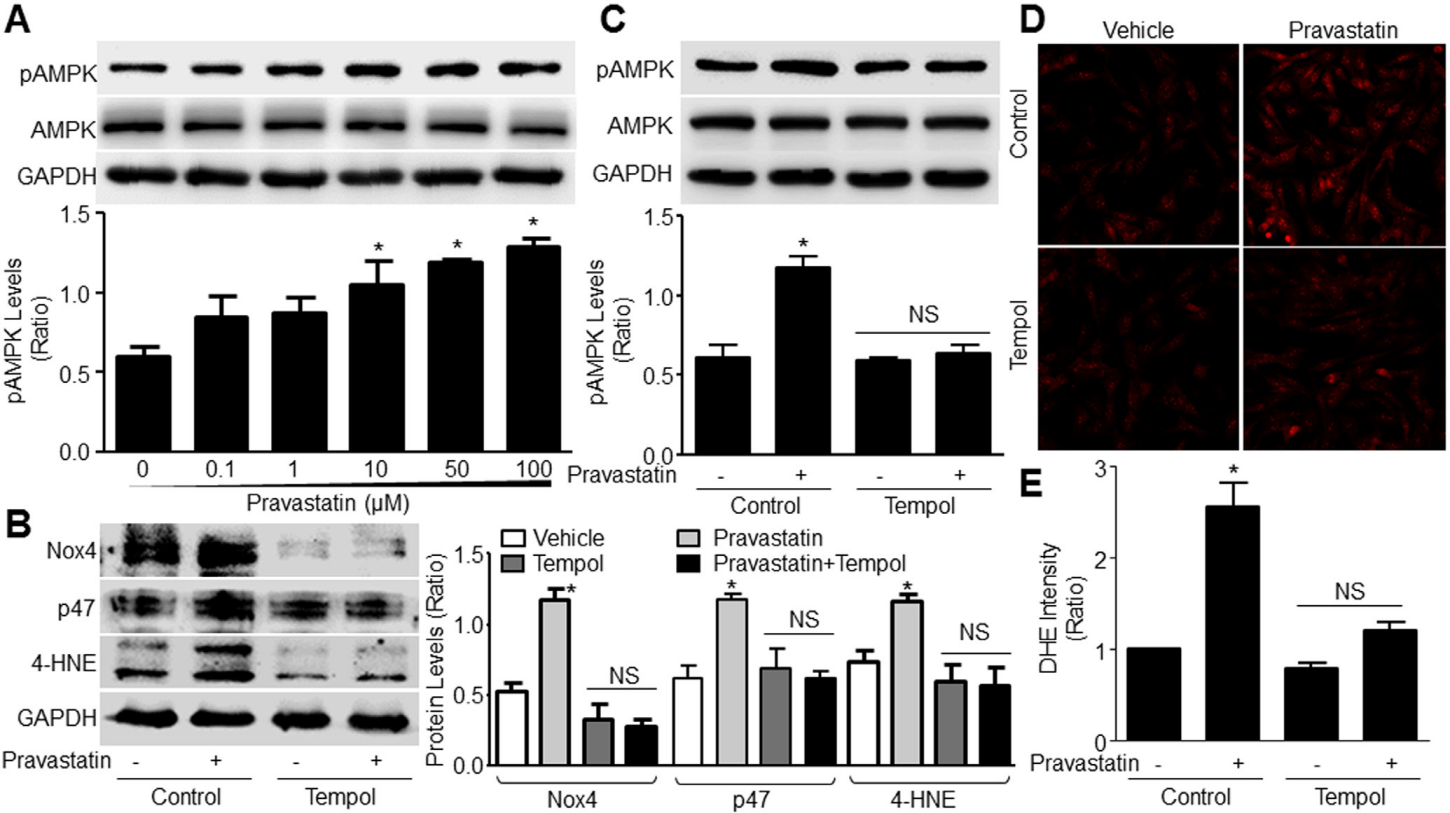
Pravastatin *via* ROS activates AMPK in murine VSMCs **A**. Murine VSMCs were treated with pravastatin for 2 hours as indicated concentrations. Total cell lysates were subjected to perform western blot analysis of the levels of pAMPK-T172. The blot is a representative of three blots from three independent experiments. **B**.-**E**. Cultured VSMCs were pretreated with tempol (10 μM, 30 minutes) followed by co-incubation of pravastatin (50 μM) for 2 hours. Total cell lysates were subjected to perform western blot analysis of the levels of **B**. oxidative stress biomarkers of Nox4, p47 and 4-HNE and **C**. pAMPK-T172. ROS productions were determined by measuring DHE fluorescence in **D**. and performed quantitative analysis in **E**. N is 3 in each group. One-way ANOVA followed by Tukey *post-hoc* tests was used for multiple comparisons. In A, *P < 0.05 *vs*. point 0. In **B**., **C**. and **E**., *P < 0.05 *vs*. Control. NS indicates no significance.

We previously reported that AMPK is activated by oxidants via threonine 172 phosphorylation (pAMPK-T172) and AMPK activation instigates AAA formation [13, 21]. Then we investigated whether pravastatin via oxidant activated AMPKα2. As indicated in Figure 2A-2C, the levels of pAMPK-T172 were gradually increased by pravastatin in concentration-dependent manner in cultured murine VSMCs. Tempol, as a SOD mimic, abolished the augments of Nox4, p47, 4-HNE, and pAMPK-T172 induced by pravastatin. Similarly, the ROS productions, assayed by determining the intensity of DHE fluorescence, were increased by pravastatin, which were abolished by tempol (Figure 2D and 2E). Taking all data together, it indicates that pravastatin via induction of oxidative stress activates AMPKα2 in VSMCs.

### Pravastatin via ROS enhances AP-2α phosphorylation in VSMCs

**Figure 3:**
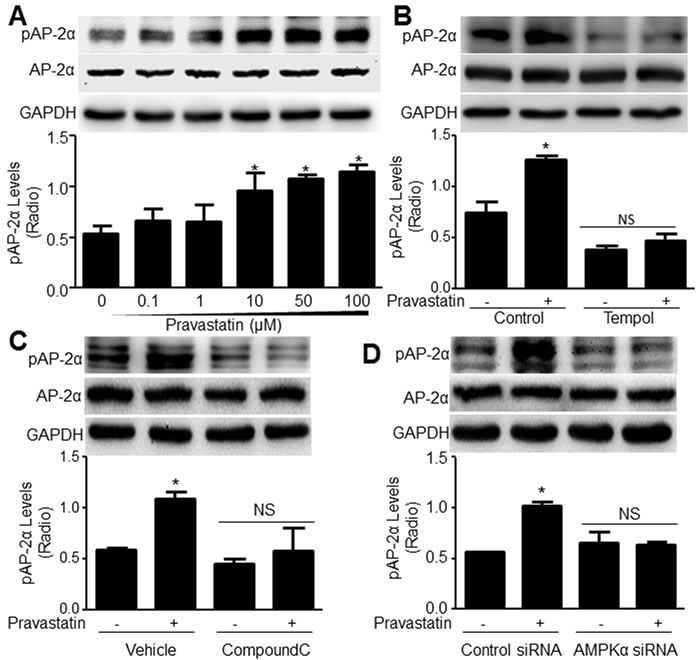
Pravastatin *via* AMPK activation increases AP-2α serine 219 phosphorylation in murine VSMCs **A**. Mouse VSMCs were treated with pravastatin for 2 hours as indicated concentrations. **B**. Cultured VSMCs were pretreated with tempol (10 μM, 30 minutes) followed by co-incubation of pravastatin (50 μM) for 2 hours. **C**. VSMCs were pretreated with compound C (20 μM, 30 minutes) followed by co-incubation of pravastatin (50 μM) for 2 hours. **D**. VSMCs transfected with AMPKα2 siRNA for 48 hours were incubated with pravastatin (50 μM) for 2 hours. Total cell lysates in **A**.-**D**. were subjected to perform western blot analysis of the level of pAP-2α-S219. N is 3 in each group. One-way ANOVA followed by Tukey *post-hoc* tests was used for multiple comparisons. In A, *P < 0.05 vs. point 0. In **B**.-**D**., *P < 0.05 vs. Control (Vehicle) or control siRNA alone. NS indicates no significance.

We have identified AP-2α as a downstream of AMPKα2 via phosphorylation at serine 219 (S219), which represents its transcriptional activity [13]. Thus, we detected whether pravastatin increases AP-2α S219 phosphorylation. As depicted in Figure 3A, pravastatin did not increase the level of pAP-2α-S219 at a concentration of 0.1-1 μM; however, at 10 μM, pravastatin significantly enhanced AP-2α S219 phosphorylation. Increasing concentrations of pravastatin (50-100 μM) further enhanced AP-2α phosphorylation. Further, reduction of ROS by tempol abolished pravastatin-induced AP-2α S219 phosphorylation (Figure 3B), revealing that pravastatin is a potential activator of AP-2α through ROS.

### AMPKα2 is involved in pravastatin-increased AP-2α phosphorylation

To determine the role of AMPKα2 in pravastatin-activated AP-2α, we pretreated VSMCs with compound C (20 μM) which functions as an AMPK inhibitor (Supplementary Figure S1A). As presented in Figure 3B, pravastatin (50 μM) for 2 hours enhanced AP-2α phosphorylation in vehicle-treated cells, but not in VSMCs pretreated with compound C.

The effects of compound C on AP-2α phosphorylation were further confirmed by transfecting VSMCs with AMPKα2 siRNA to inhibit AMPKα2 expression (Supplementary Figure S1B). AMPKα2 siRNA, but not control siRNA, blocked the effects of pravastatin on AP-2α phosphorylation (Figure 3C), suggesting that the pravastatin-increased AP-2α phosphorylation is AMPKα2 dependent.

### Pravastatin via AMPKα2/AP-2α signaling increases MMP2 gene expressions in VSMCs

**Figure 4:**
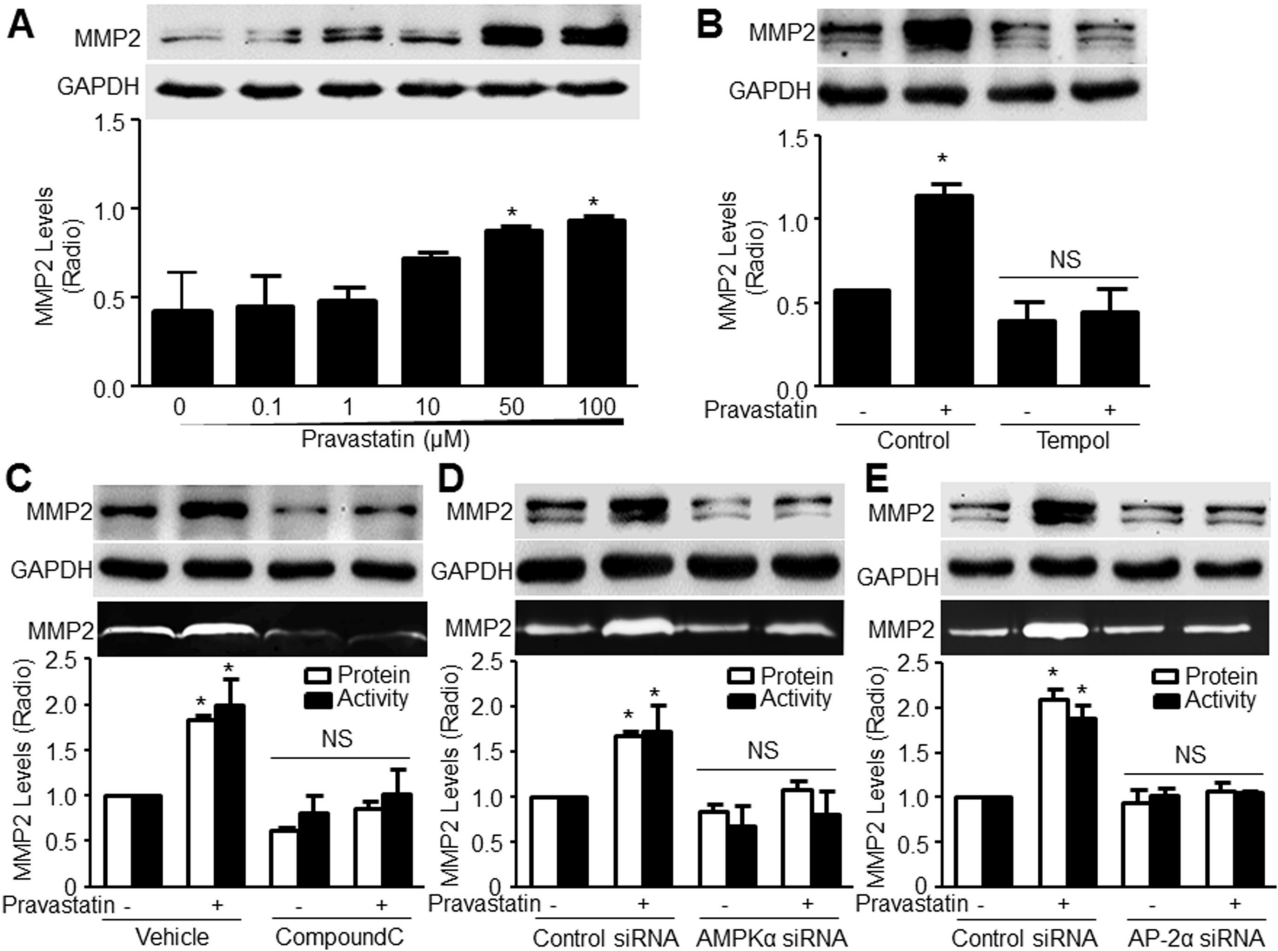
Pravastatin increases the levels of MMP2 protein and activity in murine VSMCs, which depends on AMPK and AP-2α **A**. Mouse VSMCs were treated with pravastatin for 24 hours as indicated concentrations. **B**. Cultured VSMCs were pretreated with tempol (10 μM, 30 minutes) followed by co-incubation of pravastatin (50 μM) for 24 hours. **C**. VSMCs were pretreated with compound **C**. (20 μM, 30 minutes) followed by co-incubation of pravastatin (50 μM) for 24 hours. **D**. and **E**. Cultured VSMCs transfected with **D**. AMPKα2 siRNA or **E**. AP-2α siRNA for 48 hours were incubated with pravastatin (50 μM) for 24 hours. Total cell lysates in **A**.-**E**. were subjected to perform western blot analysis of the level of MMP2 proteins. The activity of MMP2 in culture medium in **C**.-**E**. was assayed by zymography. The N is 3 in each group. One-way ANOVA followed by Tukey *post-hoc* tests was used for multiple comparisons. In **A**., *P < 0.05 vs. point 0. In **B**.-**E**., *P < 0.05 vs. Vehicle or control siRNA alone. NS indicates no significance.

To validate how activation of AP-2α by pravastatin increases the AAA formation, we detected the levels of MMP2, which is a key contributor for elastin degradation in the process of AAA and a target of AP-2α in endothelial cells [17]. In Figure 4A, pravastatin concentration-dependently increased the protein levels of MMP2 in VSMCs. As expected, either tempol (Figure 4B) or compound C (Figure 4C) significantly abolished the pravastatin-enhanced the levels of MMP2 protein and activity, and mRNA (Supplementary Figure S1C).

To further confirm the essential roles of AMPKα2 and AP-2α in pravastatin-induced upregulations of MMP2, endogenous expressions of AMPKα2 and AP-2α in VSMCs were suppressed by transfection of AMPKα2 and AP-2α siRNA (Supplementary Figure S1B and S1D). Deficiency of AP-2α had no effects on pravastatin-increased AMPK phosphorylation, indicating that AP-2α is a downstream of AMPK (Supplementary Figure S1D). As shown in Figure 4D, 4E, S1E, S1F, pravastatin increased the levels of MMP2 protein, mRNA and activity in cells transfected with control siRNA. All these effects produced by pravastatin were bypassed by knockdown of AMPKα2 and AP-2α, demonstrating that both AMPKα2 and AP-2α are required for pravastatin-induced MMP2 upregulation in VSMCs.

### Pravastatin activates AMPKα2/AP-2α/MMP2 signaling in AngII-treated murine VSMCs

**Figure 5:**
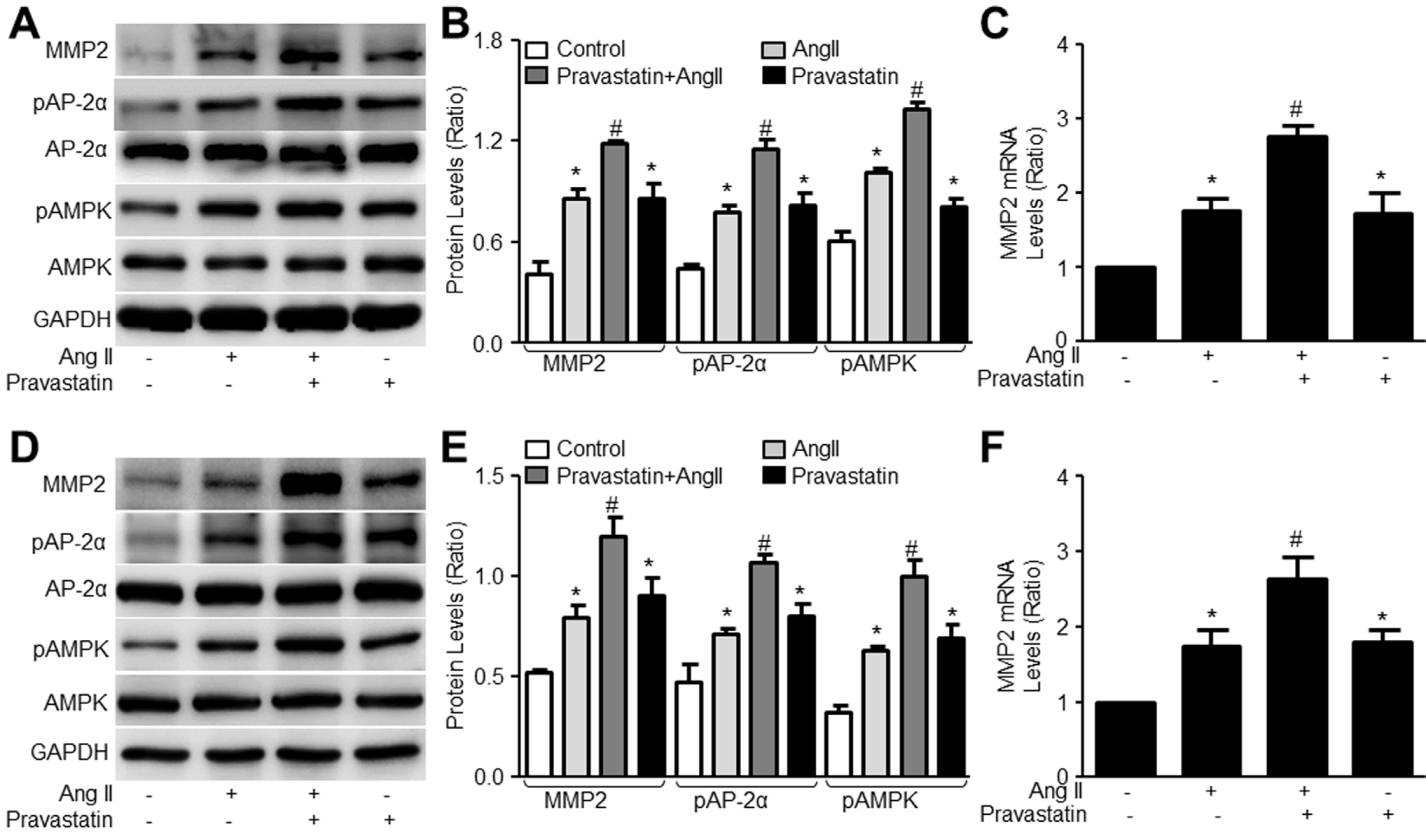
Pravastatin activates AMPK/AP-2α/MMP2 signaling in AngII-stimulated murine VSMCs **A**.-**C**. Murine VSMCs were treated with pravastatin (50 μM) for 30 minutes followed by AngII (1 μM) for 24 hours. **D**.-**F**. Human VSMCs were treated with pravastatin (50 μM) for 30 minutes followed by AngII (1 μM) for 24 hours. Cells were harvested to measure the levels of MMP2, pAMPK and pAP-2α in total cell lysates by western blot in **A**. and **D**. The quantitative analysis was shown in **B**. and **E**. The level of MMP2 was determined in **C**. and **F**. by RT-qPCR and beta-actin was used as the housekeeping gene. N is 3 in each group. One-way ANOVA followed by Tukey *post-hoc* tests was used for multiple comparisons. *P < 0.05 vs. Control. ^#^*P* < 0.05 vs. AngII alone.

Knowing that pravastatin activated the AMPKα2/AP-2α/MMP2 signaling in VSMCs under resting condition, we next examined whether pravastatin activates the AMPKα2/AP-2α/MMP2 signaling in VSMCs with AngII. As indicated in Figure 5A-5C, both AngII and pravastatin alone increased the levels of pAMPK, pAP-2α, and MMP2 protein and mRNA expressions in VSMCs. Importantly, co-incubation of pravastatin further increased the levels of pAMPK, pAP-2α, and MMP2 protein and mRNA expressions in AngII-treated VSMCs, suggesting that pravastatin activates AMPKα2/AP-2α signaling in AngII-stressed cells.

### Pravastatin activates AMPKα2/AP-2α/MMP2 signaling in AngII-treated human VSMCs

To show the translational applicability of these observations from mice, we also tested whether they can be replicated in human VSMCs. Thus, primary human aortic VSMCs were incubated with AngII plus pravastatin. Similarly, pravastatin increased the levels of pAMPK, pAP-2α, and MMP2 protein and mRNA expressions in human VSMCs with or without AngII (Figure 5D-5F), implying that pravastatin activates AMPKα2/AP-2α/MMP2 signaling in humans.

### Pravastatin induces oxidative stress, activates AMPKα2/AP-2α signaling, and upregulates MMP2 gene expression *in vivo*

The effects of pravastatin on oxidative stress, AMPKα2 and AP-2α phosphorylations, and MMP2 gene expression were further investigated *in vivo*. As indicated in Supplementary Figure S2A-S2C, in AngII-infused *Apoe*−/− mice, pravastatin administration dramatically increased the levels of 4-HNE, p47, Nox4, pAMPK-T172, pAP-2α-S219, and MMP2 protein and activity, compared to vehicle-treated mice, suggesting that pravastatin induces oxidative stress and activates AMPKα2/AP-2α/MMP2 signaling *in vivo*.

### Knockdown of AMPKα2 ablates pravastatin-enhanced AAA formation in Apoe^−/−^ mice

**Figure 6:**
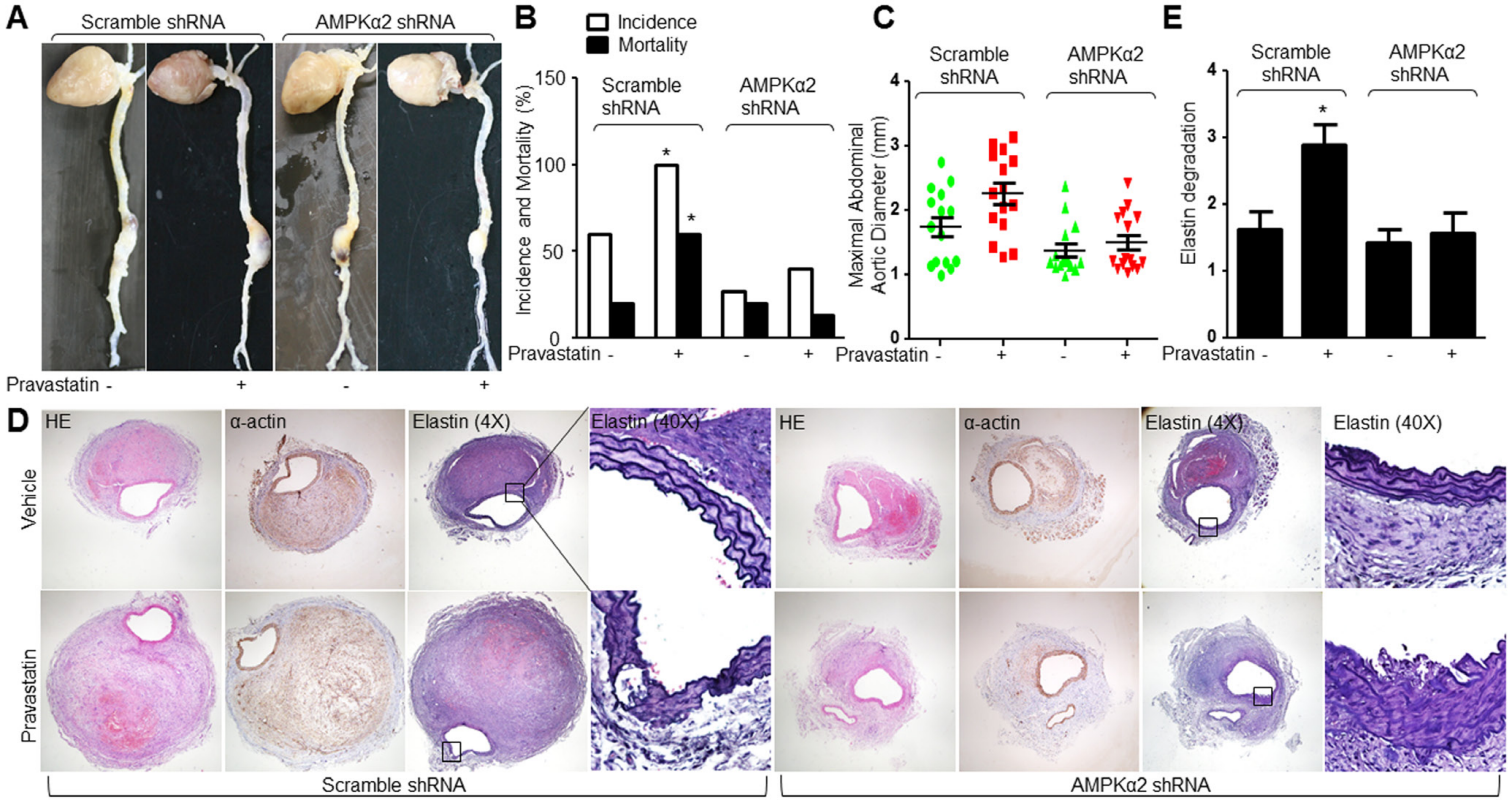
Deficiency of AMPKα2 blunts the effects of pravastatin on AAA formation in *Apoe*−/− mice The protocol and experimental designs were described in Supplementary Methods and Figure S3A. **A**. Morphological analysis of abdominal aortic aneurysm, **B**. the incidence and the mortality, **C**. maximal abdominal aortic diameter, and **D**. histological analysis of HE staining, α-actin, and Verhoff-Van Gieson staining for elastin. **E**. Grades of elastin degradation in aneurysm tissues. 15 mice in each group. Chi-Square test was used for statistical comparisons in **B**. One-way ANOVA followed by Tukey *post-hoc* tests was used for multiple comparisons in **C**. and **E**. *P < 0.05 *vs*. Scramble shRNA alone.

The role of AMPKα2 in pravastatin-increased AngII-induced AAA formation was also determined in *Apoe*−/− mice. Lentivirus-mediated RNA interference significantly inhibited AMPKα2 protein expression in abdominal aortic artery (Supplementary Figure S3B-S3E), but not affected hemodynamic parameters or metabolic indexes in AngII-infused *Apoe*−/− mice (Supplementary Table S3 and S4). Similarly, pravastatin significantly promoted the AngII-induced AAA formation in *Apoe*−/− mice infected with lentivirus expressing negative control shRNA, but not in *Apoe*−/− mice infected with lentivirus containing AMPKα2 shRNA (Figure 6A-6E). Collectively, it indicates that pravastatin via AMPKα2 activation promotes AAA formation in mice.

### Lentivirus-mediated gene knockdown of AP-2α abolishes the effects of pravastatin on AAA formation in *Apoe*^−/−^ mice

**Figure 7:**
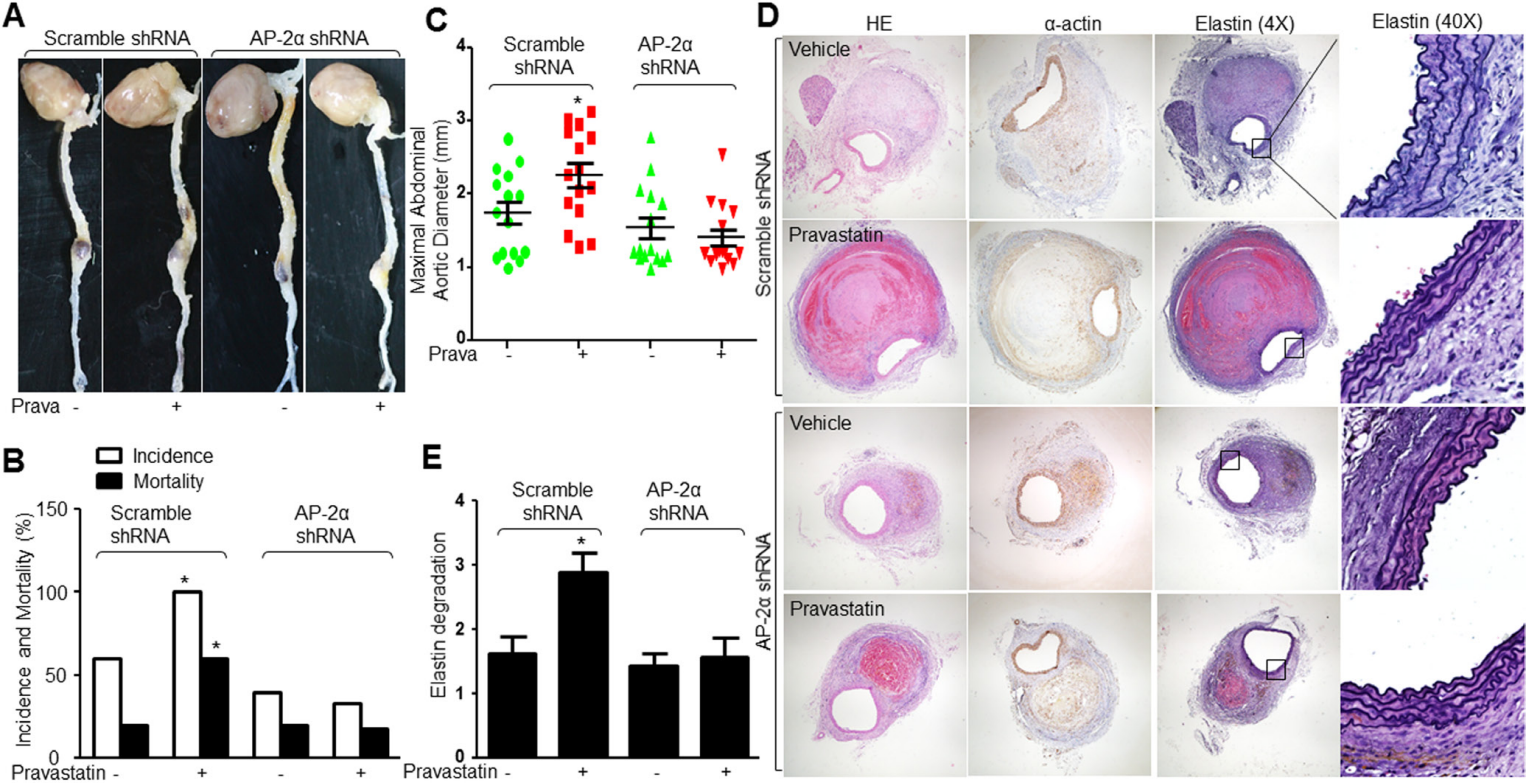
Lentivirus-mediated gene knockdown of AP-2α abolishes the effects of pravastatin on AAA formation in *Apoe*−/− mice The protocol and experimental designs were described in Supplementary Methods and Figure S3A. **A**. Representative images showing macroscopic features of abdominal aortic specimens. **B**. The incidence and the mortality and **C**. maximal abdominal aortic diameter in all AngII-infused mice. **D**. Representative histological analysis of HE staining, IHC analysis of α-actin, Verhoff-Van Gieson staining for elastin in abdominal aortic cross-section. **E**. Grades of elastin degradation in aneurysm tissues. 15 mice in each group. Chi-Square test was used for statistical comparisons in **B**. One-way ANOVA followed by Tukey *post-hoc* tests was used for multiple comparisons in C and E. *P < 0.05 *vs*. Scramble shRNA alone.

We next examined the roles of AP-2α in the effects of pravastatin on promoting AAA formation in *Apoe*−/− mice. Due to the *lethal* phenotype of mice deficient in AP-2α [16], we generated AP-2α-knockdown mice by infecting lentivirus containing AP-2α short hairpin RNA (shRNA) to downregulate AP-2α protein expression in *Apoe*−/− mice (Supplementary Figure S3B-S3E), which did not affect hemodynamic parameters or metabolic indexes in AngII-infused *Apoe*−/− mice (Supplementary Table S5 and S6). As depicted in Figure 7A, pravastatin treatment significantly accelerated the AngII-induced AAA formation in *Apoe*−/− mice infected with lentivirus expressing scramble shRNA. The incidence and mortality of AAA (Figure 7B), maximal diameter (Figure 7C), and elastin degradation (Figure 7D and 7E) in abdominal aortic artery were increased, compared to vehicle-treated mice. However, all these effects induced by pravastatin were limited in *Apoe*−/− mice if they were infected with lentivirus expressing AP-2α shRNA. Together, these data demonstrate that AP-2α is required for pravastatin-enhanced AAA formation in mice.

### AMPKα2 and AP-2α are crucial for pravastatin-induced MMP2 gene expression *in vivo*

The pathophysiological feather of AAA is the elastin degradation in vascular medium, which is mediated by MMPs (MMP2 and MMP9) [22]. We then examined the effects of AMPKα2 or AP-2α shRNA on MMP2/9 *in vivo*. As shown in Supplementary Figure S4A-S4B and S5A-S5D, the levels of MMP2 mRNA, protein, and activity were remarkably increased in *Apoe*−/− mice expressing scramble shRNA by pravastatin administration, compared to vehicle treated mice. However, either AP-2α or AMPKα2 shRNA inhibited the enchantment of MMP2 induced by pravastatin in AngII-infused *Apoe*−/− mice. The levels of MMP9 protein in cross-sections of the abdominal aortas were comparable among each group, indicating that MMP2 plays a dominant role in pravastatin-induced elastin degradation, similar to the report from Bradford C Berk's lab [23]. Taken these data together, it reveals that AMPKα2 or AP-2α is crucial for pravastatin-enhanced MMP2 activation *in vivo*.

### Pravastatin increases AP-2α and AMPKα2 phosphorylations in human subjects

**Figure 8:**
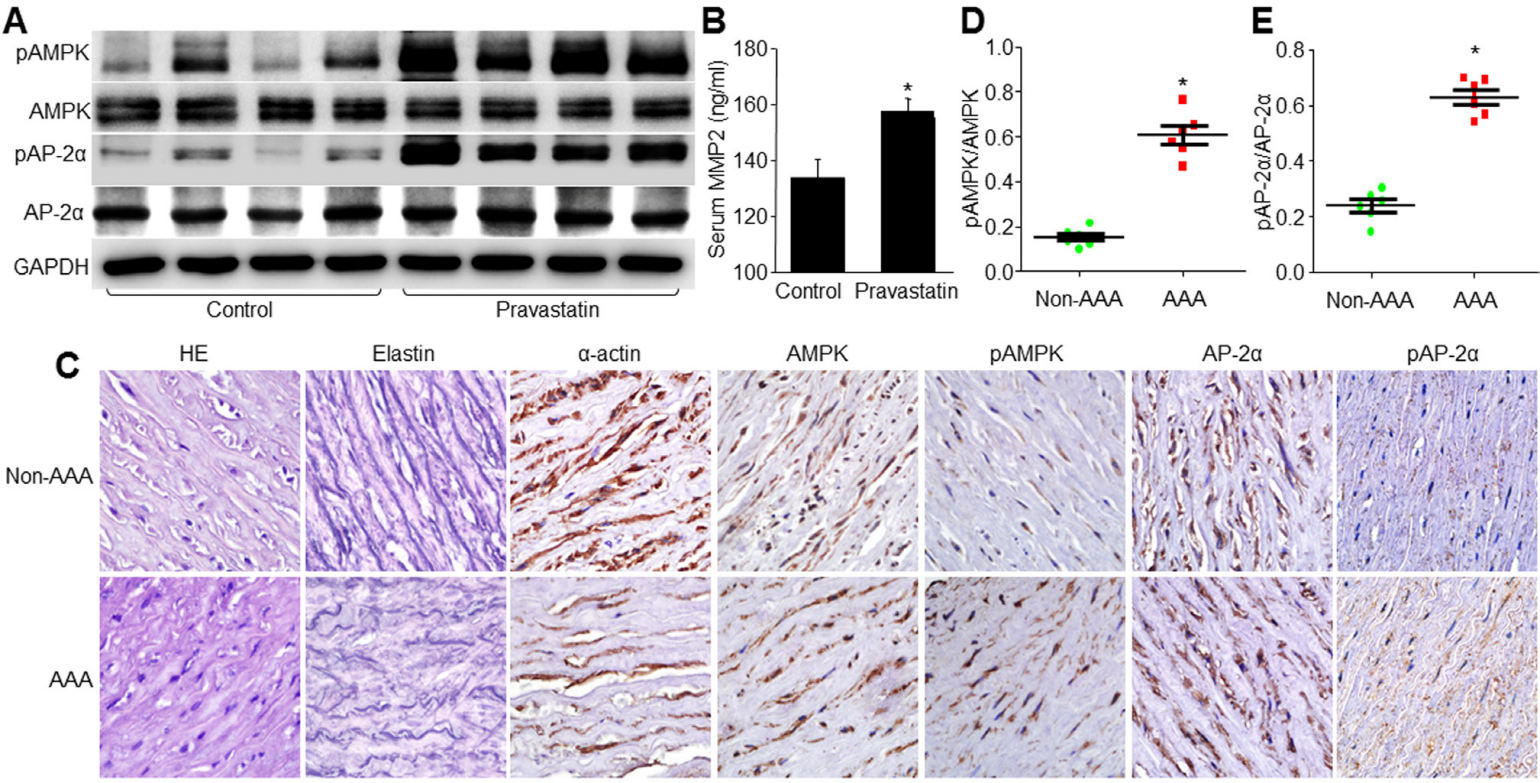
The levels of pAMPK and pAP-2α are increased in human individuals taking pravastatin and in AAA patients **A**. and **B**. The profiles of these patients were shown in Supplementary Table S9. The peripheral blood cells were collected from human subjects with or without taking pravastatin (20 mg per day) for 0.5-3 years. The levels of pAMPK-T172 and pAP-2α-S219 in total cell lysates were detected by Western blot **A**. and serum levels of MMP2 were determined by ELISA **B**.. **C**.-**E**. The demographic data were presented in Supplementary Table S10. The levels of pAMPK and pAP-2α were assayed by IHC **C**.. The quantitative analyses of pAMPK **D**. and pAP-2α **E**. from C were shown. 8 human subjects in each group in A and B. 6 human subjects in each group in **C**.-**E**. Unpaired student's *t*-test was used for all statistical comparisons. *P < 0.05 *vs*. Control patients **B**. or Non-AAA patients **D**., **E**.

To address if pravastatin at conventional dose (20 mg/day) activates AMPKα2/AP-2α signaling, we assayed the phosphorylated levels of AP-2α and AMPKα2 in peripheral blood leucocytes from human individuals taking pravastatin (Supplementary Table S7). In Figure 8A, the levels of pAP-2α-S219 and pAMPK-T172 were remarkably increased by taking pravastatin in humans, accompanied with the enhancement of serum MMP2 levels (Figure 8B). These data indicate that pravastatin is able to activate AMPKα2/AP-2α/MMP2 signaling in humans at regular dose.

### Increased AP-2α and AMPKα2 phosphorylations in AAA patients

Finally, to establish human relevance, we conducted a pilot experiment by collecting samples of AAA from human subjects (Supplementary Table S8). As shown in Figure 8C-8E, human AAA samples exhibited higher levels of AMPKα2 and AP-2α phosphorylations in aortic tissues from AAA than those from non-AAA control patients. Although the pilot experiment did not establish the cause-effect relation among AP-2α and AAA in clinical investigations, it still implies the important role of AP-2α in the process of AAA.

## DISCUSSION

The major finding of this study is that pravastatin promotes the AAA formation. To date, the effects of statins on AAA remain controversial. Simvastatin has been reported to inhibit AAA formation induced by angiotensin II (AngII) in apolipoprotein E knockout (*Apoe*−/−) mice [24–26], while rosuvastatin and atorvastatin had no benefit for abdominal aortic aneurysms [27]. Pravastatin produces bidirectional effects on cerebral aneurysm in estrogen-deficient rats, which is associated with the dose [23]. Karrowni et al retrospectively found that statin therapy led to decreased AAA growth rate in humans, and while meta-analyses and individual studies have failed to show benefit or shown mildly reduced growth [28]. Hurks et al reported that there was a trend towards increased aortic MMPs and protease activity in AAA patients taking pravastatin, but not atorvastatin or simvastatin [29]. The distinct effects of statins on AAA may depend on the subtypes, doses, durations, and AAA models.

We also identified a novel molecular mechanism that AP-2α S219 phosphorylation is crucial for pravastatin-promoted AAA formation. As a transcription factor, AP-2α recognizes the consensus DNA sequence of 5′-GCCNNNGGC-3′, which regulates a number of genes including MMP2, p21, Bcl-2, and VEGF, which are involved in multiple cell functions. We previously identified AMPKα2 as an upstream kinase of AP-2α [13]. In this study, we further discovered that statins function as AP-2α activator through AMPKα2.

The results presented in this work have for the first time established a causal effect of statins on AAA *in vivo* and uncovered the molecular mechanism by which pravastatin promotes AAA formation (Supplementary Figure S6). We suggest that pravastatin administration would be cautiously reconsidered in patients with high serum level of AngII or low blood level of apolipoprotein E, and patients with AAA.

## MATERIALS AND METHODS

Detailed information is available in Supplementary Materials.

### Animals and protocols of *in vivo* experiments

Male *Apoe*−/− mice were received pravastatin administration (50 mg/kg) in drinking water. The model of AAA was established by implanting an Alzet osmatic minipumps into *Apoe*−/− mice to deliver AngII (1.44 mg/kg/day, 28 days) as described previously [30]. Mice were injected with GFP-labeled lentivirus expressing AP-2α or AMPKα2 shRNA via tail vein as described previously [31]. At the end of experiment, all mice were sacrificed under anesthesia. The whole aortas including thoracic and abdominal aortas were collected for morphological and histological analysis of AAA.

### Analysis and quantification of AAA

To quantify AAA incidence and size, the maximum width of the abdominal aorta was measured with Image Pro Plus software (Media Cybernetics Inc.). Aneurysm incidence was defined as an external width of the suprarenal aorta that was increased by 50% or greater as described previously [32].

### Patients and sample processing

AAA tissues were obtained from human subjects under surgery. Patients were enrolled to take pravastatin (20 mg/kg/day) for 0.5-3 years. The blood was collected from human subjects before and after pravastatin treatment. Leucocytes were isolated from blood as described previously [33].

### Generation of shRNA construct and lentivirus production

Based on the protocol from Signaling Gateway, the shRNA cassette containing target sequence of AP-2α (GGAGAGCGAAGTCTAAGAATG) or AMPKα2 (GGAGAGCUAUUUGAUUAUATT) was designed. The cassette was subcloned into pEN-hH1c vector as described previously [34].

### Cell cultures

As described previously [35], mouse and human vascular smooth muscle cells (VSMCs) from ATCC were grown in basal medium (Clonetics Inc. Walkersville, MD) supplemented with 2% fetal bovine serum, penicillin (100 U/ml) and streptomycin (10 mg/ml). In all experiments, cells were used between passages 4 and 8. All cells were incubated at 37°C in a humidified atmosphere of 5% CO_2_ and 95% air. Cells were grown to 80% confluency before being treated with different agents.

### Transfection of siRNA into cells

Transient transfection of siRNA was carried out according to Santa Cruz's protocol [36].

### Detection of ROS

ROS production in culture cells was detected using the fluorescent probe DHE as described previously [36, 37].

### Gelatin Zymography

Zymography was completed by use of a MMP gelatin zymography kit (GenMed Scientific Inc., USA) as described previously [13].

### Statistical analysis

For the relative quantitation of western blot, qPCR and zymography, the intensity of bands were calculated and the background was subtracted from the calculated area. We setup the ration of control group as 1. All quantitative data were reported as mean ± SEM. One-way ANOVA followed by Tukey *post-hoc* tests was used for multiple comparisons. Chi-Square test was used for statistical comparisons of AAA incidence, mortality, and severity. Two-sided *P*-value <0.05 was considered as significant.

## SUPPLEMENTARY MATERIALS FIGURES AND TABLES


