# Supplementary material for: Pravastatin activates activator protein 2 alpha to augment the angiotensin II-induced abdominal aortic aneurysms
Source: Oncotarget. 2017 Feb 4;8(9):14294–305. doi: 10.18632/oncotarget.15104 (PMC5362406; doi:10.18632/oncotarget.15104)
Supplement: Supplementary file 1 [file oncotarget-08-14294-s001.pdf]

Pravastatin activates activator protein 2 alpha to augment the angiotensin II-induced abdominal aortic aneurysms

Supplementary Material

Contents

1. Supplementary Materials and Methods
2. Supplementary Figure Legends of S1-S6
3. Supplementary Figure S1-S6
4. Supplementary Table S1-S8

Materials and Methods

Reagents

Pravastatin was purchased from Sigma Company (Cat: 1554206). Polyclonal or monoclonal antibodies against p47, Nox4, GAPDH, α-SMA, pAMPK, AMPK, AP-2α, MMP2, MMP9, and 4-HNE were obtained from Santa Cruz Biotechnology, Abcam, or Cell Signaling Company. All secondary antibodies were from Cell Signaling Technology. Antibody of pAP-2α Serine 219 was generated by Genscript Company. Scramble siRNA, AMPKα2, and AP-2α siRNA were from Santa Cruz. Dihydroethidium (DHE) was purchased from Calbiochem (USA). Compound C (also named dorsomorphin) was purchased from Calbiochem company (Cat# 171260). Other chemicals, if not indicated, were from Sigma-Aldrich (St. Louis, MO). All drug concentrations are expressed as the final molar concentration in the working buffer.

Animals and protocols of *in vivo* experiments

Male *Apoe*$^{-/-}$ mice (8-12 weeks of age; 20-25 g body weight) were obtained from Jackson Laboratories (Bar Harbor, ME) and housed in temperature-controlled cages with a 12-h light-dark cycle and given free access to water and regular diet. The animal protocol was reviewed and

approved by the Shandong University Institute Animal Care and Use Committee.

The animal studies were consisted of three parts. In the first part, male *Apoe*$^{-/-}$ mice were received pravastatin administration (50 mg/kg) in drinking water for 4 weeks. The model of AAA was established by implanting an Alzet osmatic minipumps (Model 2004, Durect Corporation, CA, USA) into *Apoe*$^{-/-}$ mice to deliver AngII subcutaneously at a dose of 1.44 mg/kg/day or saline vehicle for 28 days as described previously[1]. AngII-infused mice were also received pravastatin administration for another 4 weeks. At the end of experiment, all mice were sacrificed under anesthesia. The whole aortas including thoracic and abdominal aortas were collected for morphological and histological analysis of AAA.

In the second or third part of the animal study, as shown in Supplementary Figure S3A, male *Apoe*$^{-/-}$ mice at age of 8-12 weeks were received pravastatin administration (50 mg/kg per day) in drinking water. After 4 weeks, mice were infected with GFP-labeled lentivirus containing scramble shRNA, AP-2α or AMPKα shRNA via tail vein injection as described previously with light modifications[2-4] and infused with AngII using an Alzet osmatic minipumps at the dose of 1.44 mg/kg/day. All mice also received pravastatin administration (50 mg/kg/day) in drinking water for 28 days. At the end of experiment, all mice were sacrificed under anesthesia. The aortas including thoracic and abdominal aortas were collected for morphological and histological analysis of AAA.

Analysis and quantification of AAA

To quantify AAA incidence and size, the maximum width of the abdominal aorta was measured with Image Pro Plus software (Media Cybernetics Inc.). Aneurysm incidence was quantified based on a definition of an external width of the suprarenal aorta that was increased by 50% or greater compared with aortas from saline-infused mice. A commonly used clinic standard to diagnose abdominal aortic aneurysm is an increase in aortic diameter of 50% as described previously[5]. The average diameter of the normal suprarenal aorta in control mice is 0.8 mm. We therefore set a threshold of 1.22 mm as evidence of an incidence of aneurysm formation.

According to the method of Daugherty et al[6], aneurysm severity was rated from none to Type IV: None: no aneurysm, Type I; lumen dilatation without

intraluminal thrombus, Type II; lumen dilatation with thrombus, Type III; conspicuous bulbous form of Type II with thrombus and multiple aneurysms with thrombus or with some aneurysms overlapping, Type IV.

Histological analysis

After hemodynamic measurements, animals were sacrificed. For morphological analysis, aortas were perfused with normal saline and fixed with 10% PBS formalin at physiological pressure for 5 minutes. The whole aortas were harvested, fixed for 24 h, embedded in paraffin, and cross-sections (5 μm) were prepared. Aortic sections of different groups were stained by hematoxylin and eosin for morphological assessment. Elastic fiber integrity of the abdominal aorta was visualized by use of a Verhoeff-Van Gieson staining kit (Gemed Scientific Inc., USA) according to manufacturer's instruction. For determination of elastin degradation, we used a standard for grades of elastin degradation as described previously[7]. Grade 1, no degradation; Grade 2, mild elastin degradation; Grade 3, severe elastin degradation; Grade 4, Aortic rupture.

Immunohistochemistry (IHC)

Formaldehyde-fixed paraffin sections were incubated with primary antibody overnight at 4ºC. The primary antibodies used were α-actin, MMP2, MMP9, 4-HNE, and P47. As a negative control, species- and isotype-matched IgG were used in place of the primary antibody. Slides were viewed with a microscope (BX41, Olympus) and with digital camera (Spot Insight 2, Diagnostic Instruments, Inc.). Because AAA lesion varies longitudinally, the suprarenal aorta was sectioned and 3 sections located at 500 μm intervals were examined. Vessel areas were measured with Image Pro Plus software (Media Cybernetics Inc.).

Immunofluorescence (IFC)

After antigen retrieval of corresponding sections on separate slides in diffirent groups, then extensive washing three times (5 min per wash) with PBS, the sections were blocked with 10% normal donkey serum in PBS and then co-incubated with the antibodies against GFP (1:100, Abcam) and AP-2α (1:100, Cell Signaling Technology, USA) or GFP (1:100, Abcam) and AMPK (1:100, Cell Signaling Technology, USA) at 4°C overnight, After washing, co-incubate with green-conjugated secondary antibody and red-conjugated

secondary antibody for 45 minutes. Take picture in fluorescence microscope.

Determination of blood pressure

Systolic blood pressures were measured in conscious mice using a noninvasive tail-cuff system with a special device designed for mice (Softron BP-98A. Tokyo, Japan). Blood pressures and heart rates were reported as mean of 3 consecutive measurements.

Serum lipid assay

Blood samples of mice were collected before euthanasia. Serum levels of total cholesterol, triglycerides, low-density lipoprotein cholesterol, high-density lipoprotein cholesterol and glucose were determined by a commercially available enzymatic assay using a biochemistry automatic analyzer (HITACHI 7170A, Hitachi, Tokyo, Japan).

Generation of shRNA construct and lentivirus production

Based on the protocol from Signaling Gateway, the shRNA cassette containing target sequence of AP-2α (GGAGAGCGAAGTCTAAGAATG) or AMPKα2 (GGAGAGCUAUUUGAUUAUATT) was designed. The cassette was subcloned into pEN-hH1c vector as described previously[8]. And then the pEN-hH1c vector containing the AP-2α shRNA or AMPK shRNA cassette was combined with an attR-containing vector pDSL-hpUP in an LR recombination reaction. The recombinant constructs of pDSL-hpUP-AP-2α-shRNA or pDSL-hpUP-AMPK-shRNA were confirmed by DNA sequence analysis. The sequence of negative control shRNA is TTCTCCGAACGTGTCACGT. The lentivirus was produced by transiently transfecting HEK293T cells using SuperFect transfection reagent (Qiagen, USA) with three packing plasmid system (pGag/Pol, pRev, and pVSV-G).The virus-containing supernatant was collected 72 hours after transfection, and filtered through 0.45 mm filters (Millipore, USA), and stored at -80°C. The titer of the viral vectors was determined by TCID50 (Tissue culture infective dose) method.

Cell cultures

Mouse and human vascular smooth muscle cells (VSMCs) from ATCC were grown in basal medium (Clonetics Inc. Walkersville, MD) supplemented with 2% fetal bovine serum, penicillin (100U/ml) and streptomycin (10mg/ml). In all experiments, cells were used between passages 4 and 8. All cells were incubated at 37°C in a humidified atmosphere of 5% CO_2 and 95% air. Cells

were grown to 80% confluency before being treated with different agents.

Transfection of siRNA into cells

Transient transfection of siRNA was carried out according to Santa Cruz's protocol[9]. Briefly, the siRNAs were dissolved in siRNA buffer (20 mM KCl; 6 mM HEPES, pH 7.5; 0.2 mM $MgCl_2$) to prepare a 10 µM stock solution. Cells grown in 6 well plates were transfected with siRNA in transfection medium (Gibcol) containing liposomal transfection reagent (Lipofectamine RNAimax, Invitrogen). For each transfection, 100 µl transfection medium containing 4 µl siRNA stock solution was gently mixed with 100 µl transfection medium containing 4 µl transfection reagent. After a 30-min incubation at room temperature, siRNA-lipid complexes were added to the cells in 1.0 ml transfection medium, and cells were incubated with this mixture for 6 h at 37°C. The transfection medium was then replaced with normal medium, and cells were cultured for 48 hours.

Real-Time Polymerase Chain Reaction

Total RNA was isolated from tissues or cells by use of Trizol reagent (Invitrogen) according to the manufacturer’s protocol. The extracted mRNA was dissolved in RNase free water, and concentrations of the total RNA were detected using a spectrophotometer. One microgram of mRNA was used for reverse transcription using PrimeCript™ RT reagent kit with gDNA Eraser (TaKaRa, Japan) containing a mixture of oligo and random primers. Amplification was performed using an iCycler iQ real-time PCR detection system. In in *vivo* or *in vitro* experiment, mRNA expression of β-actin, MMP-2 and MMP-9 was determined by means of SYBR Green technology (TaKaRa, Japan). β-actin was used as an internal control. Quantitative values were obtained from the threshold cycle value (Ct) and the $2^{-\triangle\triangle CT}$ method was used to determine relative gene expression levels. The primers of β-actin, MMP2 and MMP9 presented in as followings. β-actin, forward 5´-CACTGTGCCCATCTACGA-3´, reverse 5´-GTAGTCTGTCAGGTCCCG-3´; MMP2, forward 5´-ACCAGGTGAAGGATGTGAAGCA-3´, reverse 5´-ACCAGGTGAAGGAGAAGGCTG-3´; MMP9, forward 5´-TTGGTTTCTGCCCTAGTGAGAGA-3´, reverse 5´-AAAGATGAACGGGAACACACAGG-3´.

Western blot analysis

Total proteins were extracted from tissues or cells. Protein samples were separated on 10% SDS-PAGE and transferred to nitrocellulose membranes. After incubation in 5% skim milk for 1.5 h at room temperature, membranes were incubated overnight at 4°C with the primary antibodies including rabbit GAPDH antibody (1:1000, Cell Signaling Technology, USA), rabbit P-AMPK antibody (1:1000, Cell Signaling Technology, USA), rabbit AMPK antibody (1:1000, Cell Signaling Technology, USA), rabbit P-AP-2α Serine 219 antibody (1:1000, Genscript Company), rabbit AP-2α antibody (1:1000, Abcam), rabbit MMP-2 antibody (1:1000,Abcam), mouse MMP-9 antibody (1:1000, Abcam), rabbit 4-HNE antibody (1:1000, Abcam), rabbit Nox4 antibody (1:500, Santa Cruz Biotechnology) and rabbit p47 antibody (1:500, Santa Cruz Biotechnology). After a wash and incubation with Specific conjugated peroxidase-labeled secondary antibodies, protein bands were visualized by use of the ECL reagents (Millipore Corp., MA, USA). GAPDH expression was used to ensure equal protein loading. Protein expression levels were determined by densitometry. All experiments were repeated for three times and the mean values derived.

Detection of ROS

ROS production in culture cells was detected using the fluorescent probe DHE as described previously[9]. Briefly, before the end of treatment, 10 μM DHE was added to the medium and incubated for 30 min at 37°C, then washing with PBS twice. The DHE fluorescent intensity in cells was recorded by fluorescent reader at the wave of excitation (485 nm) and emission (645 nm). Control was setup as 1 (Ratio).

Gelatin Zymography

Zymography was completed by use of a MMP gelatin zymography kit (GenMed Scientific Inc., USA). Briefly, aortic protein extracts from mice or cell culture medium without boiling were separated by 10% SDS-PAGE polymerized in the presence of 0.1% gelatin at 4°C to detect the activities of MMP-2 and MMP-9. Gels were washed with renaturing buffer for 1 hour, and then incubated with developing buffer at 37°C for 20 hours. After incubation, gels were stained with Coomassie Brilliant Blue, and then destained with destaining buffer until clear white bands appeared on the blue background. Gel images were captured by use of a Kodak Imager (Eastman Kodak Company,

New York, USA), and the unstained, translucent digested regions represented areas of MMP activity.

ELISA

Serum from patients were collected and frozen at -80℃.Serum concentration of MMP2 was determined by using human MMP2 enzyme-link immunosorbent assay (ELISA) kit (eBioscience, USA) following the manufacturer's recommendations. All samples were measured in duplicate.

Patients and samples processing

All protocols in human studies complied with the Management Rules of the Chinese Ministry of Health and were approved by the Ethical Committee of Shandong University Qilu Hospital. For detecting the effects of pravastatin in humans, we enrolled 16 patients with hypercholesterolemia who were taking pravastatin (20 mg per day) or not. The profiles of these patients were shown in Supplementary Table S7. The blood was collected from these patients. Leucocytes were isolated from blood and subjected to Western blot analysis for the expression and phosphorylation of AMPKα2 and AP-2α.

Abdominal aortic tissues were obtained from 6 control non-AAA patients after sudden death and 6 patients with AAAs who underwent surgical procedures. Abdominal aortic lesions were confirmed by aortic morphological analysis whereas control aortic tissues were found to have no aortic diseases. The demographic data were presented in Supplementary Table S8.

Statistical analysis

For the relative quantitation of western blot, qPCR and zymography, the intensity of bands were calculated and the background was subtracted from the calculated area. We setup the ration of control group as 1. Data were displayed as scatter plots and bar graphs with indication of mean ± SEM. Statistical analyses involved use of SPSS 20 (SPSS Inc, Chicago, IL). Comparisons of parameters among two groups were made by the unpaired Student's t-test and comparisons of parameters among more than two groups were made by one-way ANOVA followed by Tukey post-hoc tests. Chi-Square test was applied to comparisons of AAA incidence, mortality and severity. A value with $P<0.05$ was considered statistically significant.

Reference

1. Daugherty A, Manning MW, Cassis LA. Angiotensin ii promotes atherosclerotic lesions and aneurysms in apolipoprotein e-deficient mice. The Journal of clinical investigation. 2000;105:1605-1612
2. Jalkanen J, Leppanen P, Pajusola K, Narvanen O, Mahonen A, Vahakangas E, Greaves DR, Bueler H, Yla-Herttuala S. Adeno-associated virus-mediated gene transfer of a secreted decoy human macrophage scavenger receptor reduces atherosclerotic lesion formation in ldl receptor knockout mice. Molecular therapy. 2003;8:903-910
3. Maegdefessel L, Azuma J, Toh R, Deng A, Merk DR, Raiesdana A, Leeper NJ, Raaz U, Schoelmerich AM, McConnell MV, Dalman RL, Spin JM, Tsao PS. Microrna-21 blocks abdominal aortic aneurysm development and nicotine-augmented expansion. Sci Transl Med. 2012;4:122ra122
4. Maegdefessel L, Azuma J, Toh R, Merk DR, Deng A, Chin JT, Raaz U, Schoelmerich AM, Raiesdana A, Leeper NJ, McConnell MV, Dalman RL, Spin JM, Tsao PS. Inhibition of microrna-29b reduces murine abdominal aortic aneurysm development. J Clin Invest. 2012;122:497-506
5. Sparks AR, Johnson PL, Meyer MC. Imaging of abdominal aortic aneurysms. Am Fam Physician. 2002;65:1565-1570
6. Daugherty A, Manning MW, Cassis LA. Antagonism of at2 receptors augments angiotensin ii-induced abdominal aortic aneurysms and atherosclerosis. British journal of pharmacology. 2001;134:865-870
7. Satoh K, Nigro P, Matoba T, O'Dell MR, Cui Z, Shi X, Mohan A, Yan C, Abe J, Illig KA, Berk BC. Cyclophilin a enhances vascular oxidative stress and the development of angiotensin ii-induced aortic aneurysms. Nat Med. 2009;15:649-656
8. Godec J, Cowley GS, Barnitz RA, Root DE, Sharpe AH, Haining WN. Inducible rnai in vivo reveals that the transcription factor batf is required

to initiate but not maintain cd8+ t-cell effector differentiation. Proceedings of the National Academy of Sciences of the United States of America. 2015;112:512-517

9. Wang S, Xu J, Song P, Wu Y, Zhang J, Chul Choi H, Zou MH. Acute inhibition of guanosine triphosphate cyclohydrolase 1 uncouples endothelial nitric oxide synthase and elevates blood pressure. Hypertension. 2008;52:484-490

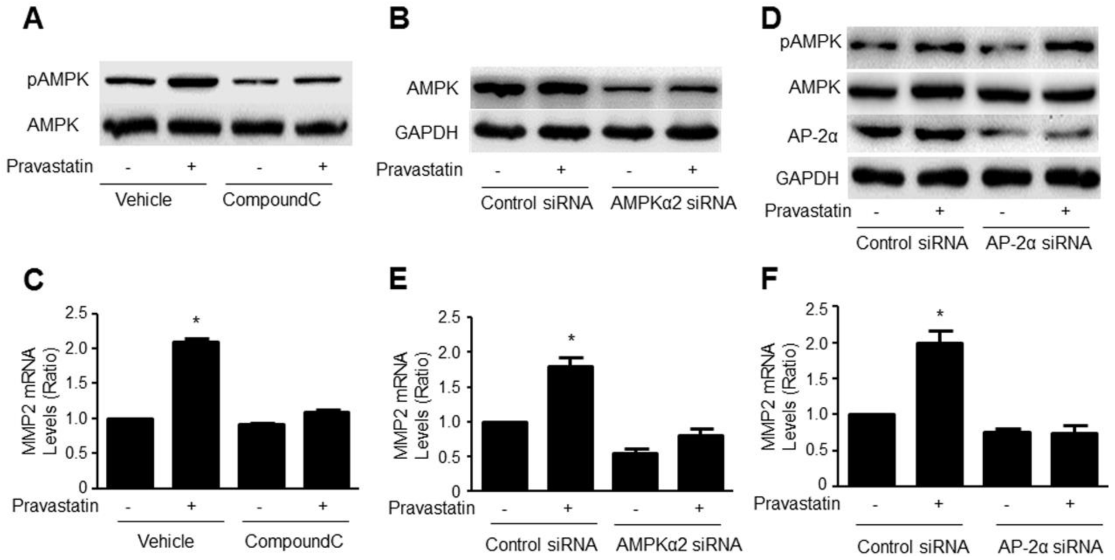

Supplementary Figure S1: Effects of Compound C, AMPKα2 siRNA, AP-2α siRNA on the levels of AMPK, AP-2α, and MMP2 in cultured VSMCs. (**A**) Cultured VSMCs were pretreated with compound C (50 μM, 30 minutes) followed by co-incubation of pravastatin (50 μM) for 2 hours. Total cell lysates were subjected to perform western blot analysis of the levels of pAMPK and AMPK. (**B**) Cultured VSMCs transfected with AMPKα2 siRNA for 48 hours were incubated with pravastatin (50 μM) for 24 hours. Total cell lysates were subjected to perform western blot analysis of the levels of total AMPK. (**C**) Cultured VSMCs were pretreated with compound C (50 μM, 30 minutes) followed by co-incubation of pravastatin (50 μM) for 2 hours. The levels of mRNA were assayed by real time PCR. *$P<0.05$ vs. Control siRNA or Vehicle.

(**D**) Cultured VSMCs transfected with AP-2α siRNA for 48 hours were incubated with pravastatin (50 μM) for 24 hours. Total cell lysates were subjected to perform western blot analysis of the levels of pAMPK, total AMPK and AP-2α. (**E**) Cultured VSMCs transfected with AMPKα2 siRNA for 48 hours were incubated with pravastatin (50 μM) for 24 hours. The levels of mRNA were assayed by real time PCR. (**F**) Cultured VSMCs transfected with AP-2α siRNA for 48 hours were incubated with pravastatin (50 μM) for 24 hours. The levels of mRNA were assayed by real time PCR. **P*<0.05 vs. Control siRNA. N is 3 in each group in all above experiments.

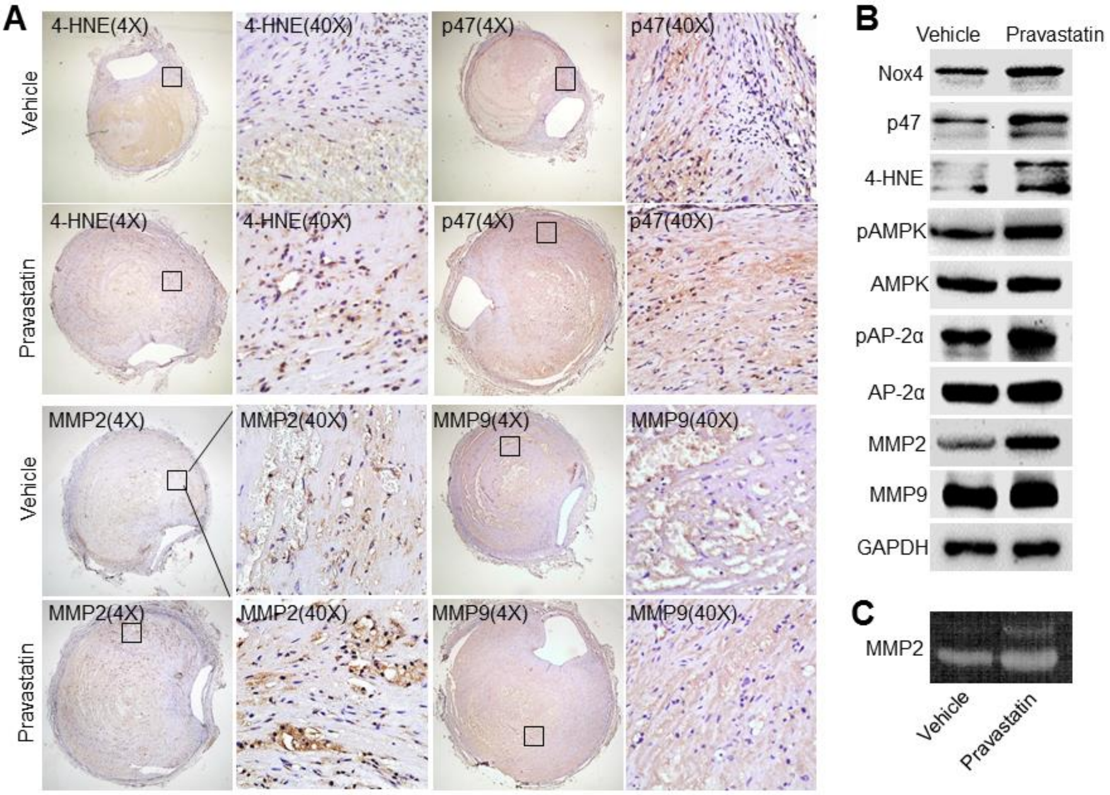

Supplementary Figure S2: Pravastatin induces oxidative stress and activates AMPKα, AP-2α, and MMP2 *in vivo*. Male *Apoe*$^{-/-}$ mice were received pravastatin administration (50 mg/kg) in drinking water for 4 weeks followed by AngII infusion (1.44 mg/kg/day) for another 4 weeks. (**A**) Representative IHC stainings of 4-HNE, p47, MMP2 and MMP9 of abdominal aortic cross-section from AngII-infused *Apoe*$^{-/-}$ mice. (**B**) Western Blotting analysis of the protein levels of 4-HNE, p47, Nox4, pAMPK, pAP-2α, MMP2 and MMP9 in homogenates of abdominal aortic arteries from AngII-infused

Apoe$^{-/-}$ mice. (**C**) MMP2 activity was determined in homogenates of abdominal aortic arteries by zymography.

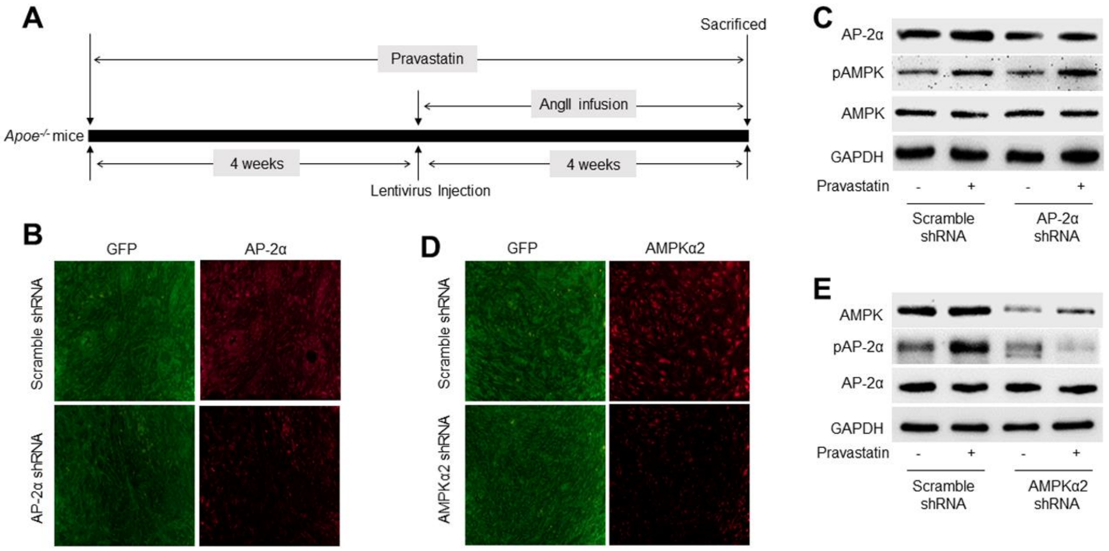

Supplementary Figure S3: The protocols of animal experiments. (**A**) The protocol of lentivirus infection, pravastatin administration plus AngII infusion in *Apoe*$^{-/-}$ mice. (**B**) Abdominal aortic arteries were isolated from mice to detect AP-2α protein expression by IFC. Green, GFP; Red, AP-2α. (**C**) Western Blotting analysis of pAMPK and total AP-2α levels in homogenates of abdominal aortic arteries isolated from mice infected with lentivirus expressing AP-2α shRNA. (**D**) Abdominal aortic arteries were isolated from mice to detect AMPKα2 protein expression by IFC. Green, GFP; Red, AMPKα2. (**E**) The levels of total AMPKα2 and pAP-2α in abdominal aortic arteries were measured by Western blotting.

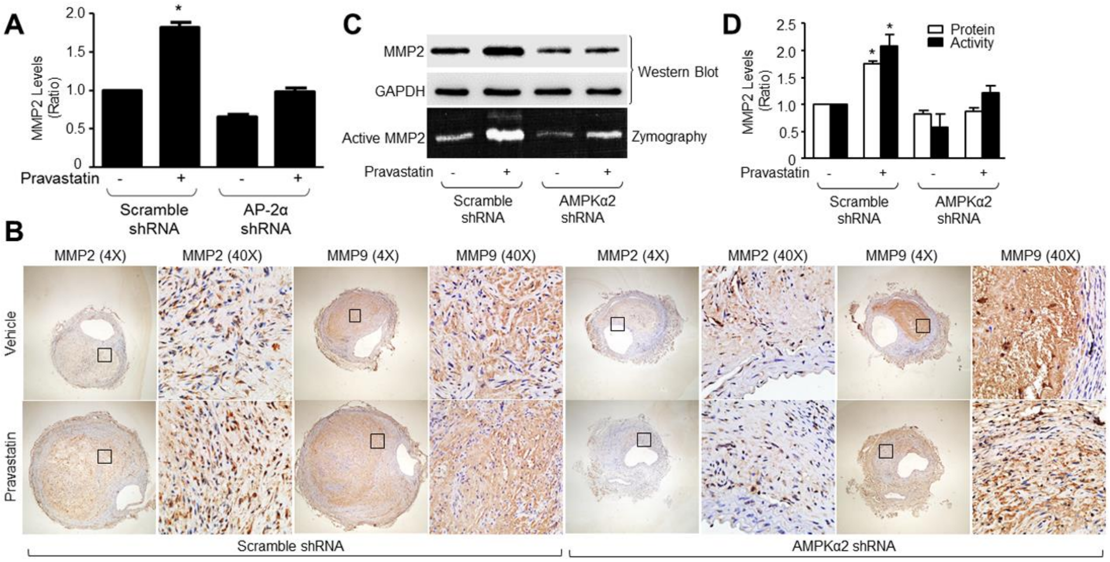

Supplementary Figure S4: Pravastatin via AMPKα2 activates AP-2α and MMP2 in *Apoe*$^{-/-}$ mice. The protocol and experimental designs were described in Supplementary Figure S3A. (**A**) The levels of mRNA were assayed by real time PCR. (**B**) IHC analysis of MMP2 and MMP9 of abdominal aortic cross-section from each group. (**C**) The levels of MMP2 protein and activity in abdominal aortic aneurysm were determined by Western blot and Zymography. (**D**) The quantitative analysis of MMP2 protein and activity in **C.** 10-15 mice in each group. *P<0.05 vs. Scramble shRNA.

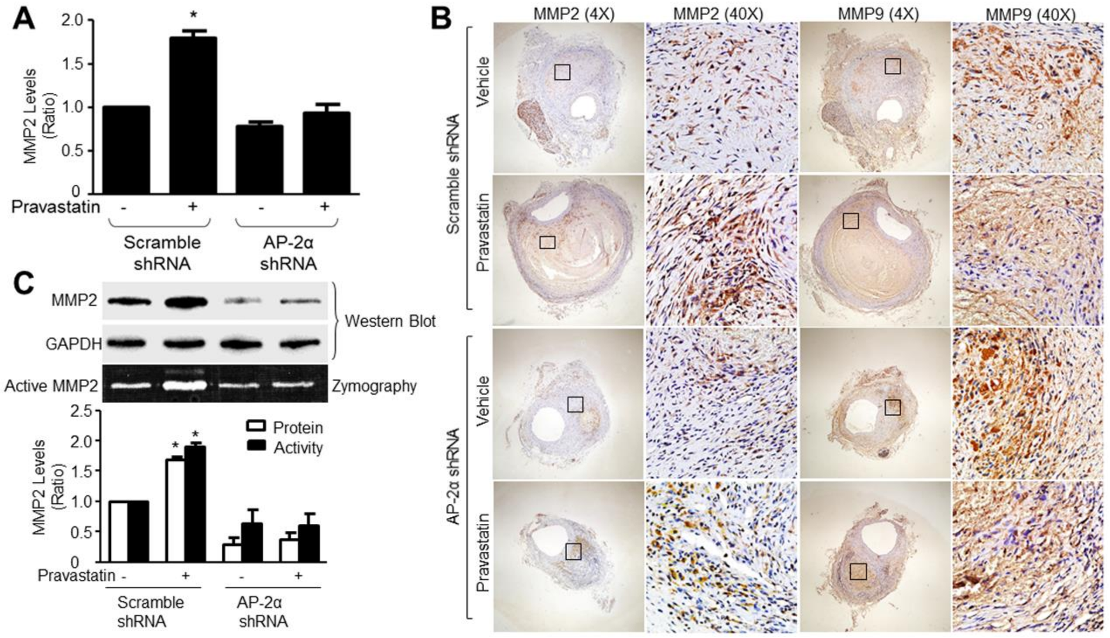

Supplementary Figure S5: Deficiency of AP-2α abolishes the effects of pravastatin on MMP2 expression in *Apoe*$^{-/-}$ mice. The protocol and experimental designs were described in Supplementary Figure S3A. (**A**) The levels of mRNA were assayed by real time PCR. (**B**) IHC analysis of MMP2 and MMP9 of abdominal aortic cross-section from each group. (**C**) MMP2 protein by Western blot and activity by zymography in abdominal aortic aneurysm from AngII-infused *Apoe*$^{-/-}$ mice. 10-15 mice in each group. *P<0.05 vs. Scramble shRNA.

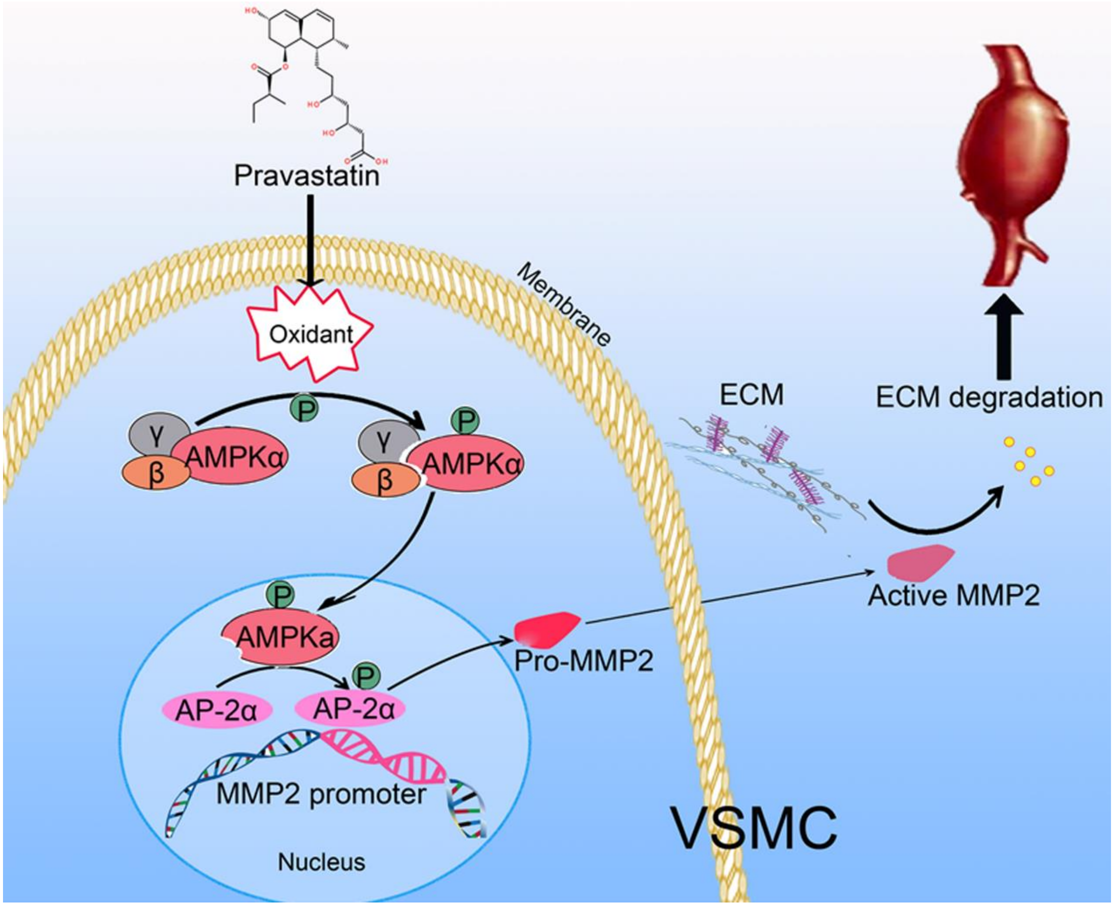

Supplementary Figure S6: The proposed mechanism by how pravastatin promotes AAA formation. Pravastatin induces oxidative stress to increase AMPK-dependent AP-2α S219 phosphorylation, resulting in the activation of AP-2α transcriptional activity in VSMCs. This leads to the upregulation of MMP2 gene expression and increased ECM degradation, causing aortic aneurysm formation.

Supplementary Table S1: Blood pressures and heart rates in AngII-infused mice

Groups	N	sBP(mmHg)	dBP(mmHg)	HR(Beats/min)
Vehicle	23	151.2 ± 5.3	92.4 ± 2.7	644.4 ± 14.0
Pravastatin	23	140.7 ± 6.6	90.3 ± 6.3	509.0 ± 24.9

sBP, systolic blood pressure; dBP, diastolic blood pressure; HR, heart rate.

Data were expressed by mean ± s.e.m.

Supplementary Table S2: Serum lipids and glucose levels in AngII-infused mice

Groups	N	TC(mmol/L)	TG(mmol/L)	LDL-C(mmol/L)	HDL-C(mmol/L)	GLU(mmol/L)
Vehicle	23	11.5 ± 1.66	1.13 ± 0.28	1.93 ± 0.30	3.25 ± 0.49	6.49 ± 0.30
Pravastatin	23	22.6 ± 1.88*	0.98 ± 0.15	6.18 ± 0.24*	2.95 ± 0.48	6.18 ± 0.24

TC, total cholesterol; TG, triglycerides; LDL-C, low-density lipoprotein cholesterol;

HDL-C, high-density lipoprotein cholesterol; GLU; glucose. Data were expressed by mean ± s.e.m. *P<0.05 vs. Vehicle.

Supplementary Table S3: Blood pressures and heart rates in AngII-infused *Apoe*$^{-/-}$ mice infected with lentivirus-AMPKα2 shRNA

Groups	N	sBP (mmHg)	dBP (mmHg)	HR (Beats/min)
Scramble shRNA	15	209.7 ± 12.9	120.7 ± 10.7	621.6 ± 12.5
Scramble shRNA + Pravastatin	15	213.0 ± 10.6	129.7 ± 14.8	513.3 ± 17.5
AMPKα2 shRNA	15	174.7 ± 17.1	113.1 ± 10.3	542.8±28.6
AMPKα2 shRNA + Pravastatin	15	200.8 ± 6.15	127.3 ± 3.67	599.1±18.2

sBP, systolic blood pressure; dBP, diastolic blood pressure; HR, heart rate. Data were expressed by mean ± s.e.m.

Supplementary Table S4: Serum lipids and glucose levels in AngII-infused *Apoe*$^{-/-}$ mice infected with lentivirus-AMPKα2 shRNA

Groups	N	TC (mM)	TG (mM)	LDL-C (mM)	HDL-C (mM)	GLU (mM)
Scramble shRNA	15	10.7 ±1.35	1.74 ± 0.22	1.24 ± 0.20	4.14 ± 0.37	6.80 ± 0.63
Scramble shRNA + Pravastatin	15	16.9 ± 0.61*	1.07 ± 0.09	2.81 ± 0.39*	5.23 ± 0.23	5.76 ± 1.28
AMPKα2 shRNA	15	13.1 ± 0.89	1.71 ± 0.17	1.66 ± 0.17	5.09 ± 0.33	5.60 ± 0.55
AMPKα2 shRNA + Pravastatin	15	13.6 ± 1.42	0.93 ± 0.06	1.79 ± 0.23	4.26 ± 0.31	6.30 ± 0.35

TC, total cholesterol; TG, triglyceride; LDL-C, low-density lipoprotein cholesterol; HDL-C, high-density lipoprotein cholesterol; GLU; glucose. Data were expressed by mean ± s.e.m. *P<0.05 vs. Scramble shRNA.

Supplementary Table S5: Blood pressures and heart rates in AP-2α-knockdown AngII-infused *Apoe*$^{-/-}$ mice

Groups	N	sBP(mmHg)	dBP(mmHg)	HR(Beats/min)
Scramble shRNA	15	209.7 ± 12.9	120.7 ± 10.7	621.6 ± 12.5
Scramble shRNA + Pravastatin	15	213.0 ± 10.6	129.7 ± 14.8	513.3 ± 17.5
AP-2α shRNA	15	201.6 ± 4.97	107.8 ± 5.82	509.9 ± 35.1
AP-2α shRNA + Pravastatin	15	198.8 ± 8.23	117.8 ± 5.38	572.2 ± 29.8

sBP, systolic blood pressure; dBP, diastolic blood pressure; HR, heart rate. Data were expressed by mean ± s.e.m.

Supplementary Table S6: Serum lipids and glucose levels in AP-2α-knockdown AngII-infused *Apoe*$^{-/-}$ mice

Groups	N	TC(mM)	TG(mM)	LDL-C(mM)	HDL-C(mM)	GLU(mM)
Scramble shRNA	15	10.7 ± 1.35	1.74 ± 0.22	1.24 ± 0.20	4.14 ± 0.37	6.80 ± 0.63
Scramble shRNA + Pravastatin	15	16.9 ± 0.61*	1.07 ± 0.09	2.81 ± 0.39*	5.23 ± 0.23	5.76 ± 1.28
AP-2α shRNA	15	10.7 ± 1.55	1.96 ± 0.28	1.24 ± 0.22	4.27 ± 0.50	5.69 ± 0.61
AP-2α shRNA + Pravastatin	15	11.6 ± 1.49	0.88 ± 0.08	1.48 ± 0.27	3.89 ± 0.30	8.16 ± 0.87

TC, total cholesterol; TG, triglyceride; LDL-C, low-density lipoprotein cholesterol; HDL-C, high-density lipoprotein cholesterol; GLU; glucose. Data were expressed by mean ± s.e.m. *P<0.05 vs. Scramble shRNA.

Supplementary Table S7: Demographic data for control individuals and patients taking pravastatin

ID	Gender	Ages (years old)	Pravastatin (dose and time)
1	Male	50	None
2	Male	71	None
3	Female	57	None
4	Female	54	None
5	Male	77	None
6	Female	62	None
7	Male	44	None
8	Female	68	None
9	Male	74	20 mg/day for 2 years
10	Female	63	20 mg/day for 1 years
11	Male	61	20 mg/day for 1 years
12	Female	54	20 mg/day for 6 months
13	Male	60	20 mg/day for 1 years
14	Male	57	20 mg/day for 2 years
15	Male	78	20 mg/day for 3 years
16	Female	48	20 mg/day for 6 months

Supplementary Table S8: Demographic data for individuals with or without AAA

ID	Gender	Ages (years old)	AAA
1	Female	48	No
2	Male	56	No
3	Female	40	No
4	Male	33	No
5	Male	60	No
6	Female	52	No
7	Male	68	AAA
8	Male	43	AAA
9	Female	59	AAA
10	Male	41	AAA
11	Male	60	AAA
12	Male	43	AAA
